# Identification of colon tumor marker NKD1 via integrated bioinformatics analysis and experimental validation

**DOI:** 10.1002/cam4.4224

**Published:** 2021-09-21

**Authors:** Yue Wang, Chunxia Yang, Wenjing Li, Ying Shen, Jianzhong Deng, Wenbin Lu, Jianhua Jin, Yongping Liu, Qian Liu

**Affiliations:** ^1^ The Third Affiliated Hospital of Soochow University, Changzhou Jiangsu Province China; ^2^ Clinical Oncology Laboratory Changzhou Tumor Hospital Affiliated to Soochow University, Changzhou Changzhou China; ^3^ Department of Oncology Wujin Hospital Affiliated with Jiangsu University Jiangsu Province China; ^4^ Department of Oncology The Wujin Clinical College of Xuzhou Medical University Jiangsu Province China

**Keywords:** bioinformatics, colon cancer, NKD1, proliferation, WGCNA

## Abstract

**Background:**

Colorectal cancer is an important death‐related disease in the worldwide. However, specific colon cancer tumor markers currently used for diagnosis and treatment are few. The purpose of this study is to screen the potential colon cancer markers by bioinformatics and verify the results with experiments.

**Methods:**

Gene expression data were downloaded from two different databases: TCGA database and GEO datasets, which were then analyzed by two different methods (difference analysis and WGCNA method). Venn and PPI analysis obtained the potential core genes, which were then performed the GO enrichment and KEGG pathway analysis. Expressions levels of NKD1 in colon carcinoma tissues were further confirmed by immunohistochemical staining and western blot assays. Moreover, the function was measured by MTT, clone formation, and tumor transplantation experiments. Importantly, co‐immunoprecipitation, immunofluorescence, and protein stability assays were further performed to explore the underlying mechanism of NKD1 promoting cell proliferation.

**Results:**

Nine potential core genes highly expressed in colon cancer samples were screened out by bioinformatics analysis. NKD1, one of the hub genes, highly expressed in the colon carcinoma tissues could enhance the proliferation of colon cancer cells. Mechanism research demonstrated that NKD1 was essential for the combination between Wnt signalosome (DVL) and β‐catenin, and that NKD1 knockout remarkably decreased the β‐catenin expression. Immunofluorescence assays further implied that NKD1 knockout significantly inhibited β‐catenin nuclear accumulation. Importantly, the stability of β‐catenin proteins was maintained by NKD1 in the colon cancer cells.

**Conclusion:**

We believe that NKD1 well expressed in the colorectal carcinoma tissues can enhance the proliferation of colon cancer cells. Furthermore, the functions that NKD1 may have in colon cancer cells should be different from that NKD1 has played in the zebrafish. Thus, NKD1 could be a specific colorectal cancer marker.

## Graphical abstract

Bioinformatics showed that NKD1 highly expressed in the colorectal carcinoma tissues, which was confirmed by experiments. NKD1 was essential for the combination between Wnt singalosome and β‐catenin. NKD1 knockout remarkably decreased the expression of β‐catenin and notably inhibited its nuclear accumulation, which resulted in the suppression of colon cancer proliferation.

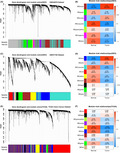

## 1 INTRODUCTION

Colorectal cancer (CRC) is one of the important death‐related diseases in the world. More than 1.8 million new cases of colorectal cancer and nearly 1 million CRC‐related deaths aroused in 2018 in the world.^1^ There are still many problems about colorectal cancer therapy needed to be solved, especially in exploring the genes critical to the proliferation of colorectal cancer cells and new effective therapeutic targets. In recent years, high throughput sequencing and microarray technology had generated a large amount of gene expression data. Thus, bioinformatics analysis could become a powerful tool to help us identify the underlying important cancer biomarkers involved in the occurrence and progression of colorectal carcinoma.^2^

NKD (naked cuticle) was firstly identified in the Drosophila.^3^ The human NKD1 (naked cuticle homolog 1) gene encodes 470‐amino‐acid polypeptide, and its NH2 domain contains the EF‐hand motif.^4^ Studies demonstrate that NKD1 inhibits the Wnt signaling pathway by preventing the β‐catenin nuclear accumulation.^5^, ^6^ The abnormal expression of NKD1 has been found in many types of tumors. Downregulation of NKD1 is related to the migration and proliferation of osteosarcoma cells.^7^ In gastric cancer cell ^8^ and non‐small cell lung cancer cells,^9^ knockdown of NKD1 enhances the cell migration and invasion. Interestingly, the NKD1 expression levels are increased in fetal kidney and the colorectal cancer cells.^4^ Moreover, study also proved that NKD1 expression was elevated in the tumors compared to the healthy tissues in the mouse model.^10^ However, the functions and involved mechanisms of NKD1 in the colon cancer cells are presently obscure.

In the present study, nine potential colorectal tumor markers (DPEP1, ARID3A, SLC5A6, AXIN2, LY6G6D, NKD1, CEL, LAPTM4B, and GRM8) were screened out from two different independent databases (TCGA database and GEO datasets) by bioinformatics analysis. We found that NKD1, one of the core genes highly expressed in the colorectal carcinoma samples, promoted the proliferation of colon cancer cells in vitro and in vivo. Further research indicated that NKD1 was essential for the combination between Wnt signalosome (Dvl) and β‐catenin. Importantly, NKD1 knockout notably decreased the β‐catenin expression and significantly inhibited its nuclear accumulation, which led to the suppression of cell proliferation. Additionally, we found that NKD1 regulated the expression of β‐catenin through maintaining its protein stability in the cells. Thus, NKD1 may be an important colorectal cancer biomarker and may function as a curative target for treatment of CRC.

## 2 MATERIALS AND METHODS

### 2.1 Collection of Data

We obtained the GSE44076 and GSE37182 datasets from the NCBI GEO Dataset (https://www.ncbi.nlm.nih.gov/geo/).^11^ GSE44076 dataset used the GPL13667 Affymetrix Human Genome U219 Array platform, which contained 148 normal colon samples and 98 colon cancer samples; GSE37182 dataset used the GPL6947 Illumina HumanHT‐12 V3.0 expression bead chip platform, which encompassed 88 normal colon tissues and 84 colorectal cancer samples. We downloaded 514 RNA transcriptomes of 41 normal colon tissues and 473 colon cancer samples from The Cancer Genome Atlas Database (TCGA) on 22 August 2020. These datasets were analyzed using R software (4.0.2 version).

### 2.2 **R software Packages**.

R software packages edgeR^12^ and limma^13^ were performed to establish the differentially expressed genes (DEGs), the pheatmap and ggplot2^14^ packages to draw the heatmap and volcano map; the packages survival^15^ and WGCNA^16^ performed the weighted gene co‐expression network analysis (WGCNA); adjusted *p* value below 0.05 and filtered logfold‐change (FC) greater than 1 were advised significant statistically in the differentially expressed gene analysis of TCGA, GSE44076 and GSE37182 datasets.

### 2.3 **DEGs and WGCNA analysis**.

The DEGs between colon cancer specimen and normal colon ones were screened by the R software packages edgeR and limma. Genes with a log FC greater than 1 and adjusted *p*‐value <.05 were advised as statistically significant DEGs. WGCNA^16^ methods analyzed the gene expression patterns of multiple samples for mining the hub genes closely related with colon cancer.

### 2.4 PPI construction

Protein–protein interaction was analyzed by the STRING website (https://www.string‐db.org/), the detail analysis methods were followed the published paper.^17^

### 2.5 Immunohistochemical staining, co‐immunoprecipitation (Co‐IP), and western blot assays

Immunohistochemical staining experiments were carried out according to the previous published paper.^18^ Anti‐NKD1 antibodies were used to incubate the slides at 4℃ for overnight. The other procedures were followed the published paper.^18^ The primary antibodies used in this study were as follows: rabbit ployclonal NKD1 antibody (ab185082, Abcam), ACTIN antibody (ab8227, Abcam), β‐catenin antibody (ab32572, Abcam), Dvl antibody (ab233003, Abcam), and Histone antibody (ab1791, Abcam). The detailed procedures were followed the published papers.^18^, ^19^

### 2.6 Generation of SW620‐NKD1^−/−^ cells

Cell lines SW620‐NKD1^−/−^ cells were generated through transfecting the SW620 colon cancer cells with the pYSY‐CMV‐Cas9‐U6‐NKD1‐sgRNA‐EFla‐neo plasmids, which were purchased from the Nanjing YSY Biotech Co. Ltd., according to the lipo2000 transfection reagent manufacture's guidance, after transfection for 2 days, the cells transfected were screened with G418 400 μg/ml relatively for 2 weeks to engender the SW620‐NKD1^−/−^ cells, western blot assays then tested the NKD1 proteins levels to evaluate the knockout efficiency.

### 2.7 Cell proliferation assays

Colon cancer cell SW620 cells were respectively seeded onto the 96 well plates (2000 cells per well) for 1 day before the transfection. 5‐Ethynyl‐2’‐deoxy Uridine (EdU) incorporation experiments were carried out to detect cell proliferation with the Cell‐Light TM EdU imaging detecting kit in accordance with manufacturer's protocols (RiboBio). We also analyzed the cell proliferation with MTT Cell Growth Assay Kits (Sigma, CT02), respectively at 0, 24, 48, and 72 h after the transfection. Moreover, clone formation experiments carried out as previously published methods.^18^ NKD1 siRNA‐1: 5'‐ACAGAAACUUGGUGGGAAATT‐3', NKD1 siRNA‐2: 5'‐CAUAAAGACAGAUGGGAAATT‐3' (Anhui, General Biosystems Co. Ltd).

### 2.8 Tumor formation assays

The animal experimental methods were according to the Guide for the Care and Use of Laboratory animals. Xenograft tumor experiments were performed in accordance with the institutional ethical guidelines. The detail practices were followed the previously published papers.^19^

### 2.9 Imunofluorescence experiments

The detailed methods were carried out according to previous procedures.^18^

### 2.10 β‐Catenin stability experiments

The colon cancer cell HCT116 cells were seeded on six‐well plates, after 5 h and then transiently transfected with empty plasmids pcDNA3.0 (1 μg) or pcDNA3‐NKD1 plasmids (1 μg) according to the lipofectamine 2000 manual. After 48 h, the cells were treated, respectively with Cycloheximide 15 μg/ml for 0, 0.5, and 1 h. β‐Catenin protein levels were measured by western blot and further analyzed by Image J software. The detailed procedures were followed the previous paper.^19^

### 2.11 Statistics

All experiments had been performed for three times. The differentially expressed genes (DEGs) were analyzed by two different analysis methods with the same criteria, a log fold‐change greater than 1 and adjusted *p*‐value below 0.05 were advised as significant statistically. We analyzed the experimental data through using unpaired two‐tailed Student's *t*‐test methods for analyzing the independent groups. The difference were advised as significant according to the *p*‐value: *: *p* < 0.05, **: *p* < 0.01, and ***: *p* < 0.001.

## 3 RESULTS

### 3.1 Analysis of differentially expressed genes

**FIGURE 1:**
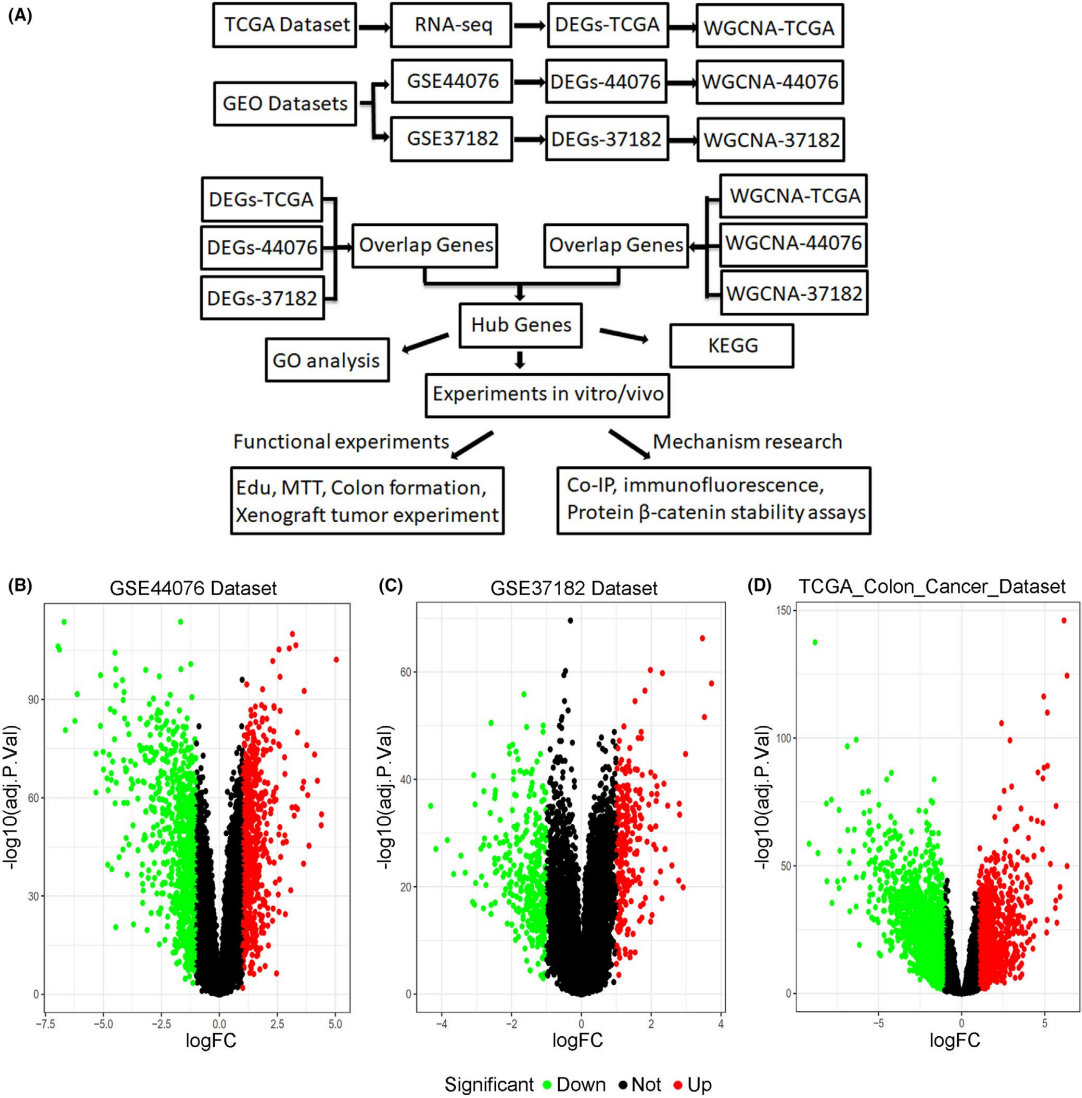
DEGs of GSE44076, GSE37182, and TCGA Colon Cancer datasets. (A) Flow chart of the present study. (B) Volcano plot of DEGs in GSE44076. (C) Volcano plot of DEGs in GSE37182. (D) Volcano plot of DEGs in the TCGA Colon Cancer dataset. Red dots mean raised genes and green dots mean declined genes, the black dots mean the genes without significant changes. The screen was performed according to the criterion: fold‐change ﹥1 and adjusted *p*‐value <0.05

To better understand this study, the flowchart was first introduced (Figure 1A). To explore the underlying colorectal carcinoma biomarkers and to avoid systematic errors, we respectively downloaded the gene expression data from two different databases, The Cancer Genome Atlas (TCGA) database and Gene Expression Omnibus (GEO) datasets. Differential expression analysis indicated that 714 increased genes and 863 decreased genes were identified from GSE44076 dataset (Figure 1B and Table S1), and 252 upregulated genes and 361 downregulated genes from the GSE37182 dataset (Figure 1C and Table S2). Moreover, the analysis of the TCGA Colon Cancer database showed 1208 upregulated genes and 2273 downregulated genes (Figure 1D and Table S3). The differentially expressed genes (DEGs) were screened with the criteria of |log2‐FC| > 1 and adjusted *p* value below 0.05.

### 3.2 WGCNA construction and key module identification

**FIGURE 2:**
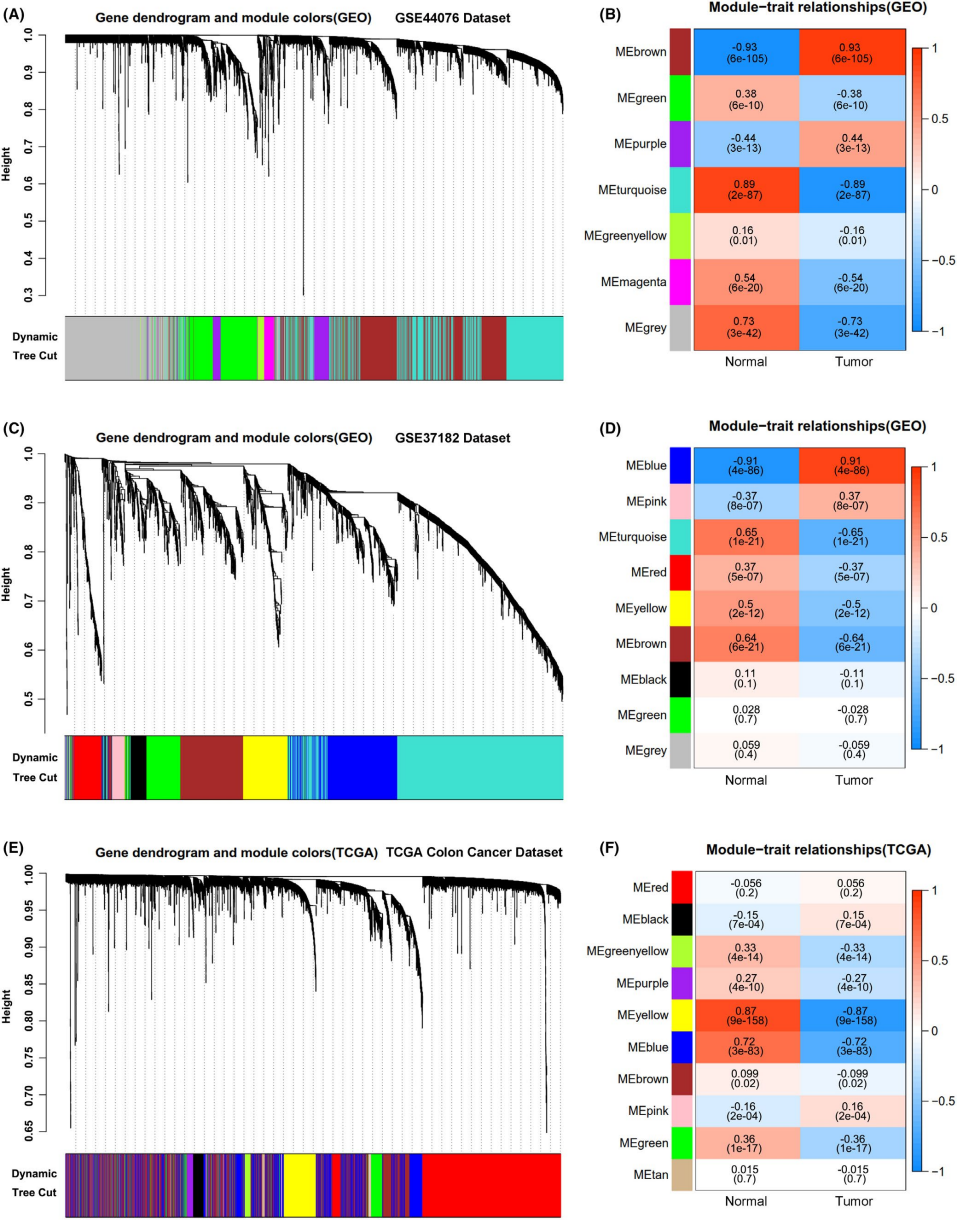
Constructions of WGCNA modules and module‐trait correlations. (A) The co‐expression modules analyzed by WGCNA and visualized in the cluster dendrogram in the GSE44076 dataset, GSE37182 dataset (C) or TCGA Colon Cancer dataset (E). Each leaf represents a separate gene and each branch represents a group of highly associated genes. (B) The module–trait relationship of genes of GSE44076 dataset, GSE37182 dataset (D) or TCGA Colon Cancer dataset (F)

To further analyze the differences of gene expression, the data from the GSE44076, GSE37182 datasets and TCGA Colon Cancer Database were further analyzed by the weighted gene co‐expression network analysis (WGCNA) method,^20^ and the results showed that seven color modules were generated from the GSE44076 dataset (Figure 2A), and the associations between the color modules and gene expression levels in the normal samples and colon tumor samples also analyzed by the WGCNA methods indicated that the genes in the brown module represented the highest expression levels in the tumor samples (0.93, *p* = 6e−105) compared to the normal samples (Figure 2B and Table S4). In addition, nine modules were produced from the GSE37182 dataset (Figure 2C), and the genes in the blue module denoted the highest expression levels in the tumor samples (0.91, *p* = 4e−86) relative to the normal samples (Figure 2D and Table S5). Moreover, 10 modules were yielded from the TCGA Colon Cancer dataset (Figure 2E), and the genes in the black and pink module indicated the highest expression levels in tumor samples (0.15, *p* = 7e−04; 0.16, *p* = 2e−04) compared to the normal samples (Figure 2F and Table S6). Overall, we analyzed the gene differential expression modules of different datasets by the WGCNA methods.

### 3.3 Identification of shared DEGs

**FIGURE 3:**
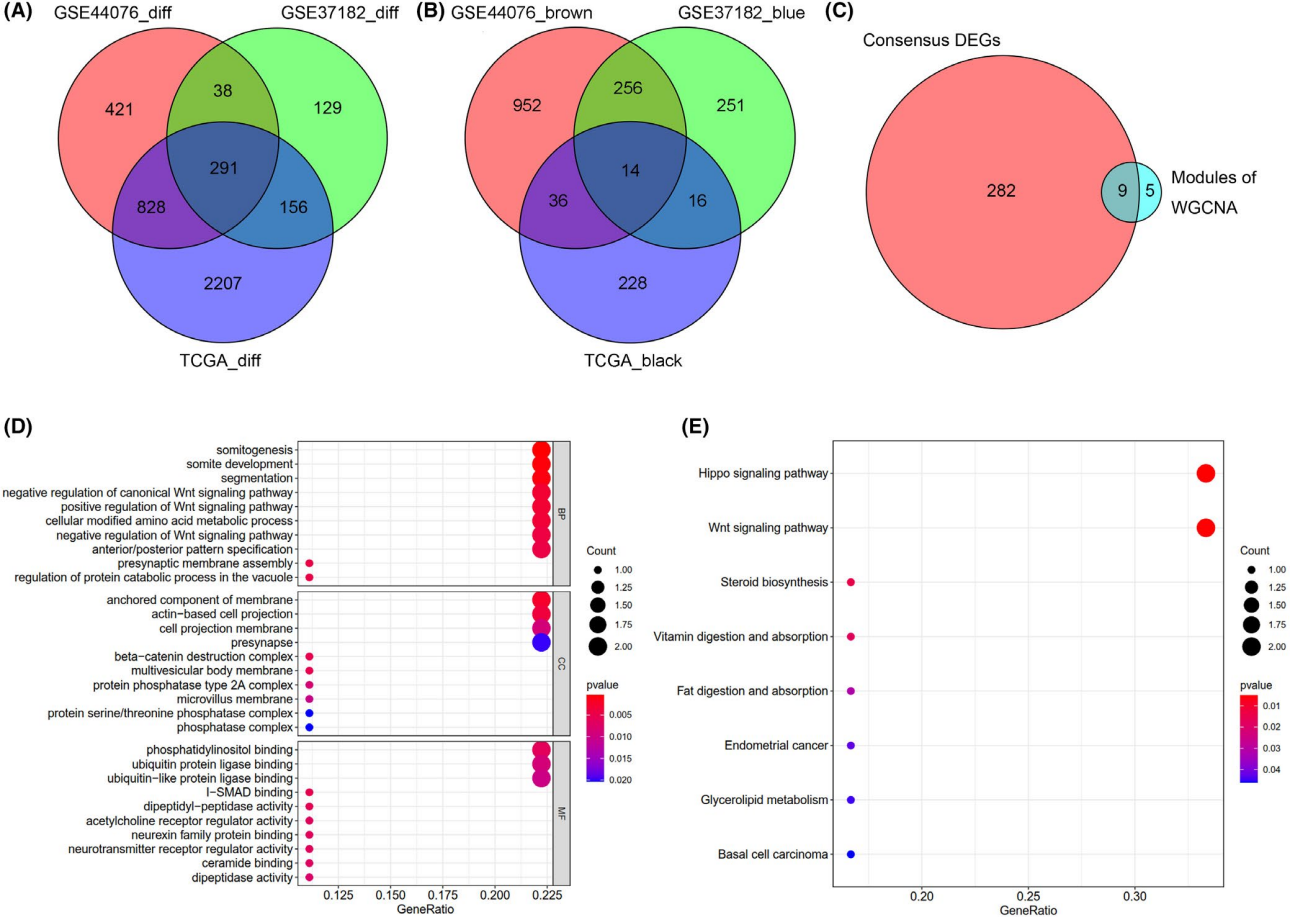
Core genes highly expressed in tumors in the three databases. (A) Venn diagrams of DEGs of GSE44076, GSE37182, and TCGA Colon Cancer datasets. In total, 291 shared genes were screened out. (B) Venn analysis of different WGCNA module genes of the three independent datasets. Gene modules highly expressed in tumors were used to screen shared genes. Fourteen shared genes of WGCNA module genes were screened out. (C) Venn analysis of the 291 shared genes of DEGs and the 14 shared WGCNA module genes. Total nine potential genes were screened out between the two different approaches of difference analysis. (D) Bubble chart of top 10 significant changes of the nine candidate genes in the GO enrichment analysis. The x‐axis means the gene numbers of different functions, y‐axis represents the top 10 different functions. The size of circle means the gene numbers of different functions, colors represent the different *p*‐values. (E) Bubble chart of top 10 KEGG pathways of the nine potential genes. The x‐axis represents the gene numbers of different signaling pathways. *p*‐value <0.05 was advised as the significant screening criteria

To further narrow the range of CRC‐related genes, Venn analysis among the DEGs of GSE44076, GSE37182, and TCGA Colon Cancer dataset were performed, a total of 291 candidate genes were commonly shared in the three independent datasets (Figure 3A and Table S7). Meanwhile, the Venn diagram also analyzed the WGCNA modules highly expressed in tumor samples, such as the brown module of GSE44076 dataset, the blue module of GSE37182 dataset, and the black module of TCGA dataset, and 14 shared genes were screened out from the three different WGCNA modules (Figure 3B and Table S8), which were further intersected with the 291 shared DEGs. Finally nine commonly shared genes (DPEP1, ARID3A, SLC5A6, AXIN2, LY6G6D, NKD1, CEL, LAPTM4B, and GRM8) were selected (Figure 3C and Table S9), implying that these nine potential core genes were likely to be the CRC‐related genes.

Subsequently, these nine potential core genes were further performed the functional enrichment analysis. Gene Ontology (GO) analysis was assigned into CC (cellular component), BP (biological process), and MF (molecular function). AXIN2 and NKD1 genes were mainly involved in the category BP included “somitogenesis,” “somite development,” “segmentation,” “negative regulation of canonical Wnt signaling pathway,” “positive regulation of Wnt signaling pathway,” “anterior/posterior pattern specification.” In CC functions, DPEP1 and LY6G6D genes were mainly involved in “anchored component of membrane,” “actin‐based cell projection,” “cell projection membrane” and NKD1 gene was involved in “protein phosphatase type 2A complex.” In MF functions, DPEP1 and LAPTM4B genes took part in “phosphatidylinositol binding,” AXIN2 and LAPTM4B genes were involved in “ubiquitin protein ligase binding” and “ubiquitin‐like protein ligase binding” (Figure 3D and Table S10). Additionally, the KEGG pathway analysis inferred that AXIN2 and NKD1 genes were mainly took part in the “Hippo signaling pathway” and “Wnt signaling pathway” (Figure 3E and Table S11).

### 3.4 NKD1 expression in the colon carcinoma specimen and cancer cells

**FIGURE 4:**
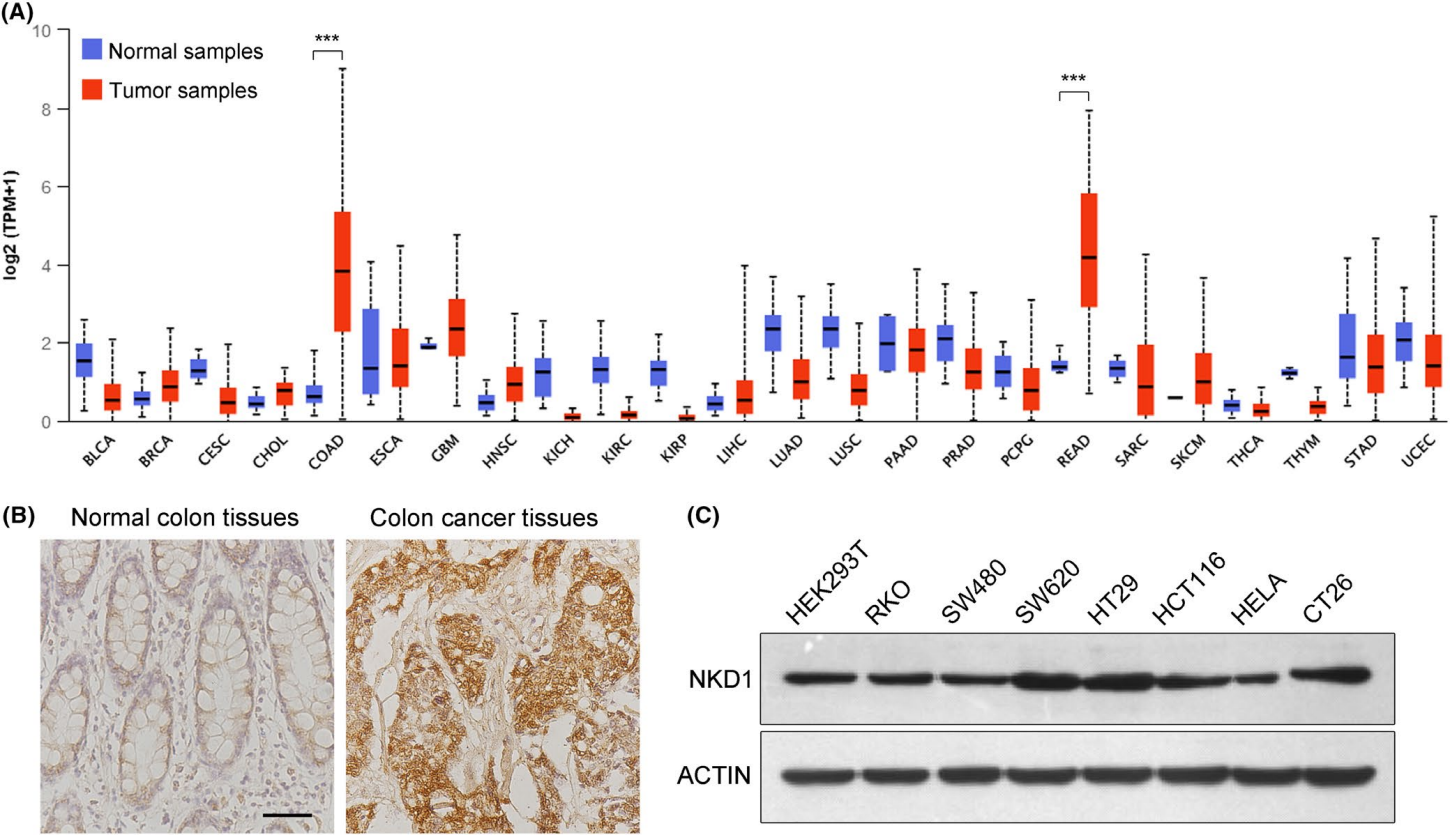
Expression levels of NKD1 in different tumor tissues and cancer cells. (A) The expression overview of NKD1 in different tumor and normal specimen across the TCGA cancers showed from the website of UALCAN. (B) NKD1 expression levels were assessed by immunohistochemical staining in the colon tumor and adjacent normal specimen, the scale bar is 50 μm. (C) The NKD1 expressions were determined by western blotting in different colon cancer cells

Through analyzing the GO functions and KEGG signaling pathways of the nine candidate core genes, we found that NKD1 acted on the Wnt signaling pathway functioned as an inhibitor. Papers reported that NKD1 was lowly expressed in many tumors.^9^, ^21^, ^22^ However, NKD1 gene highly expressed in the colon cancer samples,^10^, ^23^ which was consistent with our bioinformatics analysis results (Figures 1 and 2). To further confirm the expression levels of NKD1 in the colorectal tumor samples or cancer cells, we first analyzed the Pan‐cancer expression overview of NKD1 gene through the UALCAN web database (http://ualcan.path.uab.edu/cgi‐bin/Pan‐cancer.pl?genenam=NKD1), which showed that NKD1 was significantly downregulated in BLCA, CESC, KICH, KIRC, and so on. In addition, NKD1 highly expressed in COAD and READ (Figure 4A), which once again confirmed our bioinformatics results (Figures 1 and 2). Moreover, the immunohistochemical staining assays indicated that NKD1 expressed highly in the colon carcinoma specimen compared with the colon normal tissues (Figure 4B). NKD1 expression levels in different cancer cells inferred that NKD1 expressed in colon cancer SW620 and HT29 cells were relatively higher than that in the HEK293T, SW480, and HELA cells measured by western blot (Figure 4C). Taken together, NKD1 well expressed in colorectal tumor samples and colon cancer cells, implying that NKD1 could have important functions in the colon cancer cells.

### 3.5 NKD1 enhanced the proliferation of colon cancer cells in vitro and in vivo

**FIGURE 5:**
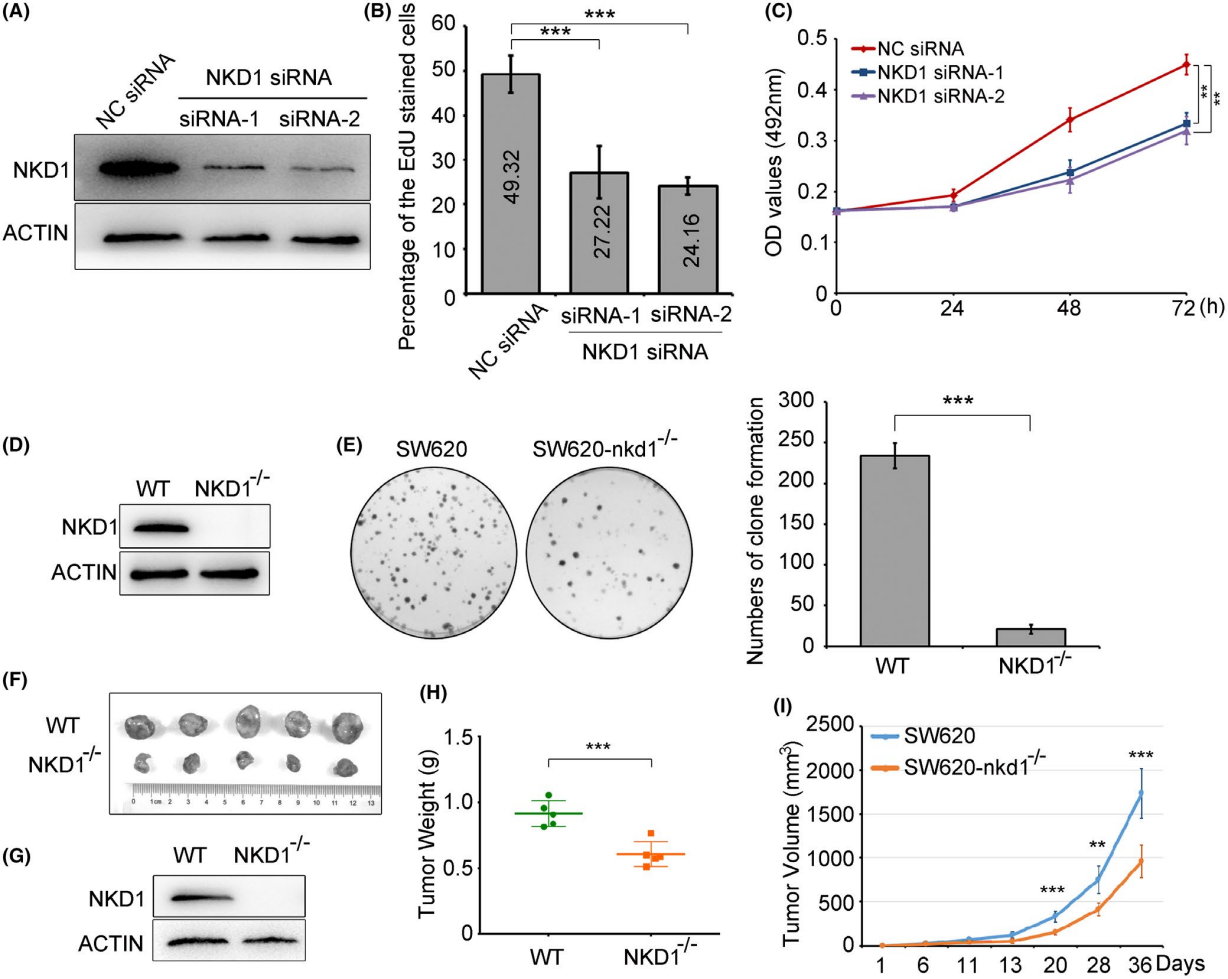
NKD1 boosts the proliferation of colon cancer cells in vitro and in vivo. (A) The efficiency of NKD1 knockdown was assessed by western blotting in SW620 colon cancer cells transfected briefly with Negative Control (NC) siRNA (100 nM), NKD1 siRNA‐1(100 nM), and NKD1 siRNA‐2(100 nM), respectively. (B) The effect of NKD1 knockdown on the proliferation of SW620 cells transiently transfected with NC siRNA (100 nM), NKD1 siRNA‐1(100 nM), or NKD1 siRNA‐2 (100 nM), were detected respectively by EdU assays or by MTT (492 nm) experiments (C). (D) NKD1 protein levels of SW620 cells and SW620‐nkd1^−/−^ cells were determined by western blot. (E) The effect of NKD1 knockout on the proliferation of SW620 colon cancer cells was tested by clone formation assays. (F) Images of dissected tumors from the nude mice injected with colon cancer SW620‐nkd1^−/−^ cells or parental SW620 (WT) cells, respectively. (G) Average protein expression levels of NKD1 from the tumor tissues were tested by western blot. (H) Diagram of the weights of tumors. (I) The growth curves of tumors transplanted with SW620 cells or SW620‐nkd1^−/−^ cells, respectively

NKD1 was highly expressed in the colon cancer SW620 cells (Figure 4C), to explore the potential functions NKD1 might have in colon cancer cells, two different NKD1 siRNA were designed and transfected into the colon cancer SW620 cells. The interference efficiency tested by western blot revealed that NKD1 expression in the cells transfected with NKD1 siRNA‐1 or siRNA‐2 was notably decreased compared with that in the cells transfected with negative control (NC) siRNA (Figure 5A). The two NKD1 siRNA were then transfected into the colon cancer SW620 cells to perform the EdU experiments and MTT assays, respectively. The numbers of EdU stained cells transfected with NKD1 siRNA‐1 or siRNA‐2 were significantly reduced relative to that of cells transfected with NC siRNA (Figure 5B), which implied that NKD1 knockdown suppressed the proliferation of colon cancer SW620 cells. MTT results also evidenced that OD values of the cells transfected with NKD1 siRNA‐1 or siRNA‐2 declined notably compared with the cells transfected with NC siRNA (Figure 5C), which suggested that NKD1 knockdown could inhibit the proliferation of colon cancer cells.

In order to further verify the proliferation function of NKD1 in vivo, the colon cancer SW620‐nkd1^−/−^ cell was constructed by knocking out nkd1 gene through Crispr Cas9 technology. We first transfected the pYSY‐CMV‐Cas9‐U6‐NKD1‐sgRNA1‐EFla‐neo plasmids into colon cancer SW620 cells, after 48 h transfection, the cells were then screened for 2 weeks by G418. We obtained the monoclonal SW620‐nkd1^−/−^ cell by infinite dilution methods. Knockout efficiency of NKD1 proteins in parental SW620 cells and SW620‐nkd1^−/−^ cells examined by western blot showed that NKD1 expression in SW620‐nkd1^−/−^ cells disappeared (Figure 5D). Clone formation assays demonstrated that the numbers of clones of SW620‐nkd1^−/−^ cells were notably reduced than that of parental SW620 cells (Figure 5E), suggesting that NKD1 knockout remarkably inhibited the growth of colon cancer cells. To further confirm the effect of NKD1 on the proliferation of colon cancer cells in vivo, the tumor transplantation assays in mice were performed. The tumors generated from SW620‐nkd1^−/−^ cells were notably smaller than those of parental SW620 (WT) cells (Figure 5F), implying that NKD1 knockout significantly restrained the proliferation of colon cancer cells. NKD1 expression in the tumors generated from the SW620‐nkd1^−/−^ cells were vanished assessed by western blot (Figure 5G). In addition, the data showed that SW620‐nkd1^−/−^ cells displayed a notable decrease in both tumor weights and tumor volumes compared with parental SW620 cells (Figure 5H,I), which implied that NKD1 knockout significantly suppressed the tumorigenesis of colon cancer cells. Taken together, inhibition of NKD1 expression significantly suppressed cancer cell proliferation both in vitro and in vivo.

### 3.6 The stability of β‐catenin proteins maintained by NKD1 in colon cancer cells

**FIGURE 6:**
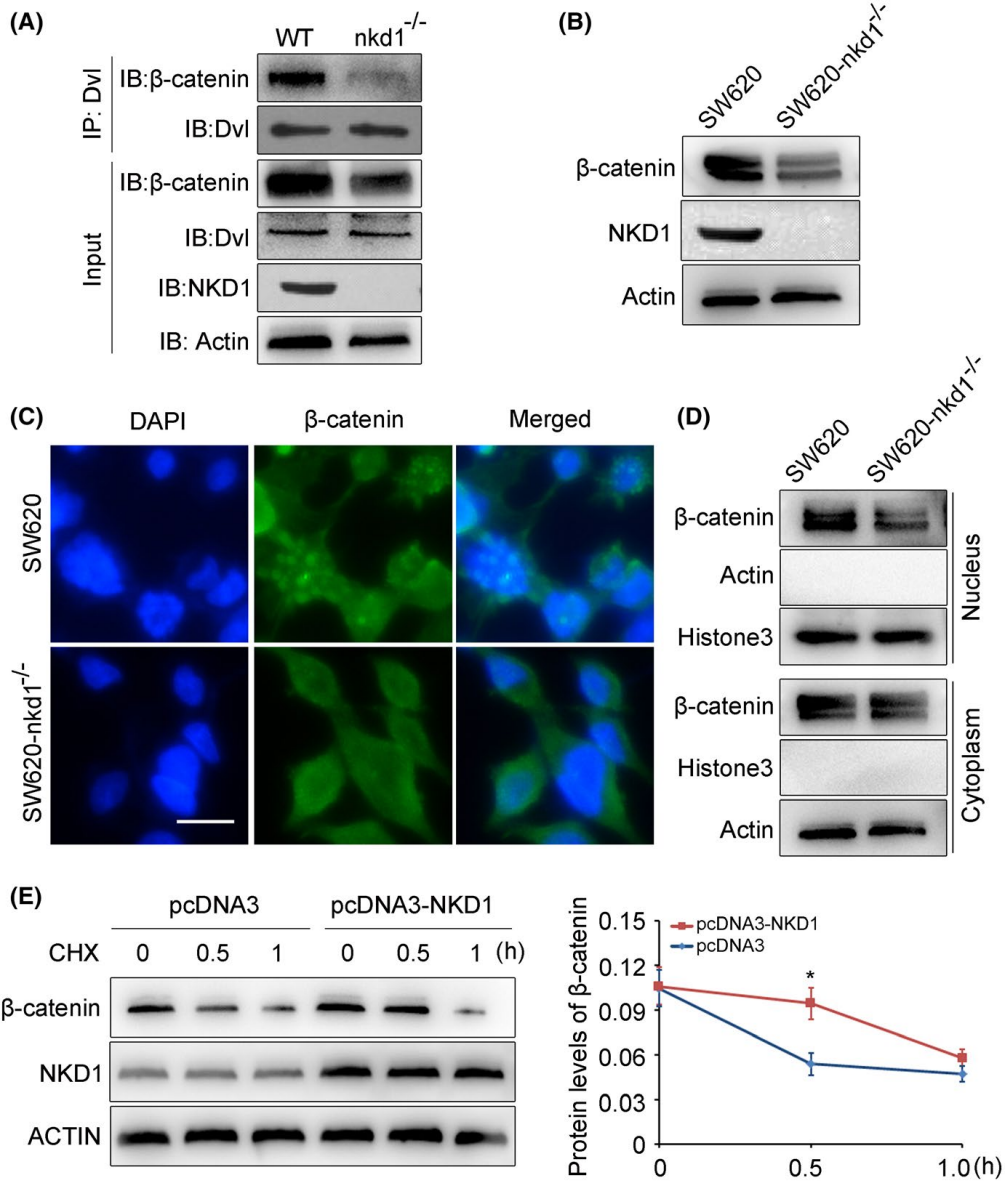
The stability of β‐catenin was retained by NKD1 in the colon cancer cells. (A) Endogenous dishevelled segment polarity protein (DVL) was immunoprecipitated with Dvl antibody and rec‐Protein A‐Sepharose, respectively from the colon cancer SW620 cells and SW620‐nkd1^−/−^ cells, and then the β‐catenin protein in precipitation was detected by western blot. (B) β‐catenin and NKD1 proteins in the SW620 colon cancer cells and SW620‐nkd1^−/−^ cells were measured by western blot, respectively. (C) The distributions of endogenous β‐catenin in SW620 cells and SW620‐nkd1^−/−^ cells were examined by immunofluorescence experiments. (D) β‐catenin expression in the nucleus or cytoplasm of colon cancer SW620 cells and SW620‐nkd1^−/−^ cells was measured, respectively by western blot. (E) The effect of CHX on the half‐life of β‐catenin proteins in the HCT116 colon cancer cells transfected briefly with pcDNA3(1 μg) or pcDNA3‐NKD1 plasmids(1 μg) for 48 h and then the cells were conducted with CHX (15 μg/ml) for different time points (0, 0.5, 1.0 h). Image J software was used to calculate the relative gray scale values of β‐catenin normalized to Actin

Papers^3^, ^5^, ^24^ reported that NKD1 functioned as an antagonist of Wnt signaling, because NKD1 recruited to the Wnt signalosome with dishevelled segment polarity protein (DVL), which inhibited the combination between Wnt signalosome and β‐catenin, causing β‐catenin proteasome degradation and preventing its nuclear accumulation,^6^ which led to the suppression of Wnt/β‐catenin signal pathway. Our previous results displayed that NKD1 had the function of promoting the proliferation of colon cancer cells, which seemed to be contradicted with the published papers. To explore the underlying mechanisms by which NKD1 involved in the Wnt/β‐catenin signal pathway in colon cancer cells, we first wondered whether NKD1 regulated the binding affinity between Dvl and β‐catenin. Co‐immunoprecipitation (Co‐IP) assays were carried out. Based on the same amount of Anti‐Dvl antibodies and rec‐Protein A‐Sepharose Beads in IP samples, we found that NKD1 knockout terminated the interaction between Dvl proteins and β‐catenin proteins, inferring that NKD1 was essential for the combination between Dvl and β‐catenin. Moreover, the Co‐IP results also showed that NKD1 knockout significantly suppressed the β‐catenin expression in the cells (Figure 6A), which implied that NKD1 knockout inhibited the Wnt/β‐catenin signal pathway. We then wondered whether NKD1 expression could regulate the expression of β‐catenin, western blot results showed that NKD1 knockout notably decreased the expression of β‐catenin in the colon cancer SW620 cells (Figure 6B). To further clarify whether NKD1 knockout modulates the nuclear accumulation of β‐catenin proteins, immunofluorescence images displayed that NKD1 knockout strikingly inhibited β‐catenin nuclear accumulation (Figure 6C). Furthermore, we extracted, respectively the cytoplasm proteins and nuclear proteins of parental SW620 cells and SW620‐nkd1^−/−^ cells, the western blot results indicated that β‐catenin expressed in the nucleus was remarkably reduced in the NKD1 knockout cells (Figure 6D), which was consistent with the immunofluorescence results (Figure 6C), these data proposed that NKD1 knockout strikingly inhibited β‐catenin nuclear accumulation, which restrained the Wnt/β‐catenin signal pathway. To investigate how NKD1 regulates β‐catenin expression, the HCT116 colon cancer cells were transfected transiently with pcDNA3 (empty plasmid) or pcDNA3‐NKD1 (plasmids expressing NKD1 proteins) for 48 h, the cells were then conducted with cycloheximide (CHX) for different time points to impede the protein translation. Degradation of the endogenous β‐catenin proteins was measured by western blot, and the gray scale calculation of protein bands was assessed by Image J software. Relative to the control cells, the half‐life of β‐catenin proteins in HCT116 cells overexpressed NKD1 was obviously prolonged (Figure 6E), signifying that NKD1 could stabilize β‐catenin in the colon cancer cells. Taken together, NKD1 knockout remarkably reduced the β‐catenin expression and inhibited its nuclear accumulation, which further inhibited the Wnt/β‐catenin signal pathway and finally suppressed the proliferation of colon cancer cells.

## 4 DISCUSSION

In recent years, researchers had discovered some potentially important colorectal tumor markers through experimental studies.^25^, ^26^ However, the current colorectal tumor markers used in clinical detection were still limited.^27^ Therefore, the aim of this study is to explore potential colorectal cancer markers through bioinformatics analysis and then followed by experiment confirmation.

In order to avoid the systematic errors, we downloaded the gene expression data from two different databases: TCGA and GEO databases. Moreover, we analyzed the gene expression data with two different methods: differential expression analysis and WGCNA method. Finally, nine potential core genes (DPEP1, ARID3A, SLC5A6, AXIN2, LY6G6D, NKD1, CEL, LAPTM4B, and GRM8) were screened out through Venn analysis and PPI construction. Some genes have been reported to be involved in the occurrence and development of colon cancer: DPEP1 promotes the proliferation of colon cancer cells via DPEP1/MYC feedback loop regulation,^19^ ARID3A promotes the development of colorectal cancer by upregulating AURKA,^28^ CDX2 inhibits the proliferation and tumor formation of colon cancer cells by transactivation of AXIN2 expression,^29^ LY6G6D significantly overexpressed (around 15‐fold) in CRC when compared with its relatively low expression in other human solid tumors.^30^ These genes that had been reported could be used as a positive control to further validate that our screening methods were feasible.

NKD1, one of the potential core genes, lowly expressed in most of the solid tumors.^7^, ^9^, ^21^ NKD1 known as passive antagonist of Wnt signaling, because NKD1's ability to antagonize canonical Wnt/β‐catenin signaling was enhanced in two different zebrafish mutant lines.^24^ In zebrafish embryo, Nkd1 binds to β‐catenin and prevents its nuclear accumulation, which was considered to be the important mechanism by which NKD1 negatively regulates the Wnt/β‐catenin signaling.^6^ However, NKD1 was well expressed in colorectal carcinoma tissues,^10^, ^31^ and the possible role of NKD1 may have in the colon cancer cells is still obscure. Paper reported that specific NKD1 mutations promote Wnt‐dependent tumorigenesis in mismatch repair deficient colorectal carcinoma.^23^ Moreover, in the intestinal tumorigenesis of two mouse models, NKD1 also highly expressed in tumors relative to the healthy tissues, which confirmed that NKD1 represented a robust marker of neoplastic growth.^10^

In the present study, we first confirmed that NKD1 was well expressed in the colon carcinoma tissues and colon cancer cells (Figure 4), which was consistent with the published paper.^10^ We then further explored the possible function of NKD1 in the colon cancer cells. To better measure the possible functions of NKD1, we generated a NKD1 knockout cell line SW620‐nkd1^−/−^ cells by Crispr/Cas9 methods. NKD1 knockdown or knockout remarkably inhibited the growth of colon cancer cells in vitro and in vivo, which were contrary to the result that NKD1 was an antagonist of the Wnt signaling pathway. We thought that this might be the specific regulation of NKD1 in colon cancer cells. Paper had reported that NKD1 bound to DVL and prevented the combination between Wnt signalosome (DVL) and β‐catenin, which resulted in the degradation of β‐catenin proteins, and further led to the suppression of cell proliferation.^5^, ^6^ We then wondered whether NKD1 affected the interaction between DVL and β‐catenin in the colon cancer cells. The Co‐IP assays showed that NKD1 knockout caused DVL and β‐catenin to no longer bind to each other, inferring that NKD1 was essential for the combination between Wnt signalosome and β‐catenin. Moreover, we also found that NKD1 knockout notably decreased the expression of β‐catenin, while the DVL expression was not affected in the cells. The results were not consistent with the results performed in the zebrafish, that is zebrafish NKD1 can promote the Dvl degradation.^32^ We thought this might be caused by the differences in species. Importantly, immunofluorescences further indicated that NKD1 knockout remarkably decreased the nuclear accumulation of β‐catenin proteins in colon cancer cells, inferring that NKD1 knockout inhibited the Wnt/β‐catenin signal, which well explained why NKD1 knockout suppressed the growth of colon cancer cells.

In conclusion, we screened the potential colorectal tumor markers from two different independent databases by bioinformatics. We found a novel function of NKD1 in promoting the proliferation of colon cancer cells, and a fresh mechanism proposing NKD1‐mediated regulation of β‐catenin durability in colon cancer cells. These discoveries provide new insights into the function and underlying mechanism of NKD1 in the colon cancer cells. Additionally, the other identified potential hub genes were also needed further experimental studies for the diagnosis, prognosis, and treatment of CRC.

## CONFLICTS OF INTEREST

The authors declare that they have no competing interests.

## ETHICS APPROVAL AND CONSENT TO PARTICIPATE

This study was approved by the Ethics Committee of Third Hospital Affiliated with Soochow University. Informed consents were obtained from the patients before the study.

## CONSENT FOR PUBLICATION

Not applicable.

## Supporting information

Table S1

Table S2

Table S3

Table S4

Table S5

Table S6

Table S7

Table S8

Table S9

Table S10

Table S11

## Data Availability

The datasets used in the current study are available from the corresponding author on reasonable request.
